# The Pathogenic Role of Smooth Muscle Cell-Derived Wnt5a in a Murine Model of Lung Fibrosis

**DOI:** 10.3390/ph14080755

**Published:** 2021-07-31

**Authors:** André Carmo-Fernandes, Michelle Puschkarow, Karin Peters, Stefanie Gnipp, Marcus Peters

**Affiliations:** 1 Department of Molecular Immunology, Ruhr-University Bochum, 44780 Bochum, Germany; andre.carmofernandes@ruhr-uni-bochum.de (A.C.-F.); karin.peters@rub.de (K.P.); 2 Department of Experimental Pneumology, Ruhr-University Bochum, 44780 Bochum, Germany; michelle.puschkarow@rub.de (M.P.); Stefanie.Gnipp@lanuv.nrw.de (S.G.)

**Keywords:** pulmonary fibrosis, bleomycin, Wingless-Type MMTV integration site family, member 5A, fibrotic foci, airway smooth muscle

## Abstract

Idiopathic pulmonary fibrosis (IPF) is a disease characterized by extensive fibrosis of the lung tissue. Wnt5a expression was observed to be upregulated in IPF and suggested to be involved in the progression of the disease. Interestingly, smooth muscle cells (SMC) are a major source of Wnt5a in IPF patients. However, no study has been conducted until now to investigate the precise role of smooth muscle-derived Wnt5a in IPF. Here, we used the bleomycin-induced lung fibrosis model in a conditional gene-deficient mouse, where the Wnt5a gene was excised from SMC. We show here that the excision of the Wnt5a gene in SMC led to significantly improved health conditions with minimized weight loss and improved lung function. This improvement was based on a significantly lower deposition of collagen in the lung with a reduced number of fibrotic foci in lung parenchyma. Furthermore, the bleomycin-induced cellular infiltration into the airways was not altered in the gene-deficient mice compared with wild-type mice. Thus, we demonstrate that the Wnt5a expression of SMC of the airways leads to aggravated fibrosis of the lung with poor clinical conditions. This aggravation was not an influence in the bleomycin-induced inflammatory processes but on the development of fibrotic foci in lung parenchyma and the deposition of collagen.

## 1. Introduction

Interstitial lung diseases are characterized by widespread inflammation and/or extensive fibrosis that mainly affects the lung parenchyma. The main problem with these diseases is that they can develop into a progressive fibrosis state with an excessive production and deposition of extracellular matrix (ECM) proteins, leading to a persistent decline in lung function. Idiopathic pulmonary fibrosis (IPF) is characterized by the progressive fibrogenesis of the lung and belongs to the family of interstitial lung diseases. Wnt5a (Wingless-Type MMTV integration site family, member 5A) was found to be highly expressed in humans who suffer from IPF [1]. It was shown here that smooth muscle cells (SMC), in addition to myofibroblasts, are a major source of Wnt5a in IPF patients. However, it is still undefined which role Wnt5a plays regarding the severity of the disease.

Wnt5a is a glycoprotein that belongs to the Wnt signaling pathway: An important pathway in the embryogenesis and development of many organs, including the lung [2], that is found unbalanced and dysregulated in many fibrotic diseases [3,4]. Wnt5a was found to be highly expressed in many diseases besides fibrotic diseases, such as cancers and lung infections like the COVID-19 disease [5].

Wnt5a belongs to the noncanonical Wnt pathway, which can activate planar cell polarity signaling and, subsequently, JNK and AP1 to stimulate cell polarity and motility [6,7]. Or it can stimulate calcium-dependent signaling that results in cell adhesion and migration through PKC upregulation [8], plus the activation of T cells and inhibition of the canonical pathway by activating the nuclear factor of activated T cells [9,10]. The activation of different pathways is determined by different receptors and microenvironments. Nonetheless, Wnt5a can also stimulate the canonical pathway by binding to frizzled receptor Fzd4 and the coreceptor LRP5 [11]. Interestingly, Fzd4 is downregulated in chronic obstructive pulmonary disease patients, preventing alveoli wound repair [12], but highly expressed in IPF patients [13]. Moreover, the high expression of TGF-β in IPF patients can induce expression of Wnt5a and its release in extracellular vesicles which can act in an autocrine manner to induce the proliferation of fibroblasts [14].

The airway SMC are one cellular source of Wnt5a. The SMC of the airways are involved in the contraction of the respiratory system, but can also be stimulated by cytokines to induce the expression of ECM proteins and other factors [15,16]. In fact, Wnt5a is the protein most expressed from its family of cytokines in SMC [17].

One way to study the mechanisms of fibrogenesis behind IPF and other fibrotic diseases of the lung in vivo is with the cytostatic drug bleomycin. Bleomycin is used against many cancers, such as cervical cancer and Hodgkin’s lymphoma, as it induces tumor cell death through the release of free radicals and DNA strand breaks [18]. However, due to the high toxicity of bleomycin in the lungs, higher dosage ultimately results in the development of lung fibrosis [19]. Due to this side effect and the fact that it is simple to administer in rodents, bleomycin is delivered to them via intranasal (i.n.) or intratracheal (i.t.) routes to induce lung fibrosis. Bleomycin-induced lung fibrosis in C57BL6 mice is characterized by an initial inflammatory phase with a high production of proinflammatory cytokines, reactive oxygen species and epithelium damage in the first fourteen days, followed by a fibrotic phase that begins from the tenth day after bleomycin administration, when the typical ECM upregulation and deposition is observed [20].

In this study, the role of Wnt5a in the development of lung fibrosis was evaluated by inducing lung fibrosis with the cytostatic drug bleomycin in conditional knockout (KO) mice lacking the Wnt5a gene in SMC.

## 2. Results

### 2.1. Airway Smooth Muscle-Derived Wnt5a Contributes to Worse Clinical Symptoms and Lung Function

**Figure 1:**
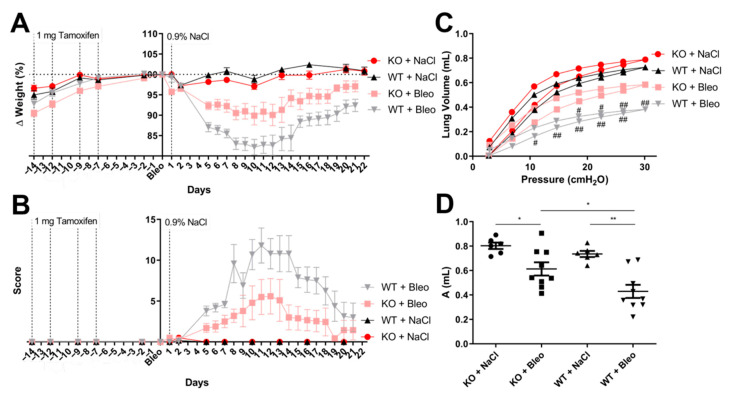
Clinical symptoms of SMC-derived Wnt5a knockout (KO) and wild-type (WT) mice after the inoculation of the saline solution or bleomycin (Bleo). (**A**) Mice weight variation through the experiment is represented in percentage values, where the day of bleomycin (Bleo) or saline solution (day 1) administration was chosen as the reference (100%). (**B**) Mice were supervised every day and a score was given according to the different clinical signs, such as weight loss, behavioral change and altered respiratory frequency. Mice were narcotized on the last day of the experiment and subjected to lung function measurements through the flexiVent system; where (**C**) the pressure–volume curve was performed in a pressure–driven way, and (**D**) the inspiratory capacity volume was obtained automatically from the flexiWare software from the pressure-volume curve. All data are shown as mean ± SEM per group, */# *p* < 0.05; **/## *p* < 0.01 by two-way ANOVA with the Bonferroni post-test (**C**) and Mann–Whitney test (**D**). Statistics between KO and WT mice in Figure C are represented with an #. *n* = 6 (KO + NaCl), *n* = 10 (KO + Bleo), *n* = 6 (WT + NaCl) and *n* = 9 (WT + Bleo). The experiment was repeated twice with similar results.

A clear weight reduction was observed upon treatment with bleomycin compared with control mice (Figure 1A). The weight loss showed a similar trend in both bleomycin treated groups. However, KO mice showed weight loss to a lesser extent than WT mice. Moreover, these weight variations were reflected in the total score obtained during the experiment, where bleomycin-treated KO mice showed overall improved health conditions throughout the experiment compared with bleomycin-treated WT mice (Figure 1B).

After the period of observation, mice were subjected to an analysis of respiratory mechanics via an invasive measurement of the lung function. A pressure–volume curve was obtained (Figure 1C) and inspiratory capacity volumes were deduced (Figure 1D). A lower inspiratory capacity and enhanced stiffness of the lung tissue was observed in control mice when compared with KO mice after bleomycin-induced lung injury. All other parameters obtained from the flexiVent system (SCIREQ) also showed a worse phenotype in the WT mice upon bleomycin administration compared with the KO line (Figure S2 in the Supplementary Materials). In addition to the inspiratory capacity volume measured from the pressure–volume curve, the static compliance and shape constant were obtained from the curve; all three parameters allow one to determine a fibrotic disease phenotype upon a decrease of these values. Here, WT mice had a lower static compliance (Figure S2A) and shape constant (Figure 1B) than KO mice. A scan based on the single compartment model was also performed, which allows one to determine the following parameters of the respiratory system: compliance—how easily the lungs can be inflated, elastance—elastic properties that the airflow needs to overcome to reach the tidal breathing of each mouse, and resistance—resistive forces against the airflow in and out the lungs. The WT mice showed difficulties regarding the lung inflation from the flexiVent system (Figure S2C), a higher elastic barrier upon airflow (Figure S2D) and more resistance against airflow (Figure S2D) compared with KO mice. There was no change in the effect of bleomycin on the resistance against the airflow in the main conducting airways—Newtonian resistance—in the model of constant phase (Figure S2F); however, a tendency was seen in the lower ability of the lungs of WT mice to retract and revert to the original shape upon forced airflow after bleomycin-induced lung fibrosis (Figure S2G) compared with KO mice, and a higher amount of energy was lost within the peripheral airways due to resistance in WT mice compared with KO mice upon lung fibrosis (Figure S2H).

### 2.2. Loss of Wnt5a Expression in Airway Smooth Muscle Cells Ameliorates Fibrogenesis

**Figure 2:**
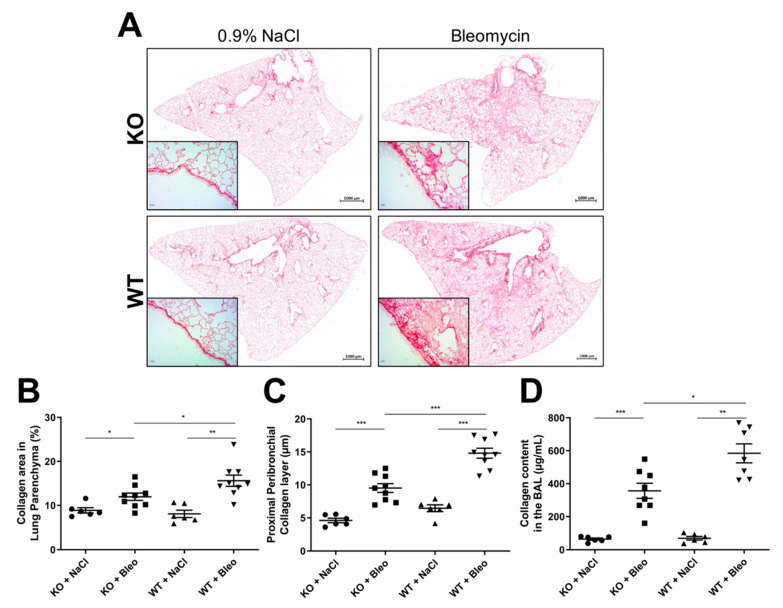
Analysis of collagen expression and deposition in bleomycin-induced lung fibrosis in KO and WT mice. (**A**) Images were recorded of the whole lung sections (scale: 1000 µm) and of proximal bronchi (scale: 500 µm) stained with Sirius red, where (**B**) the collagen area in the parenchyma was extrapolated as a percentage and (**C**) the thickness of the collagen layer in the proximal peribronchial was measured in µm. Additionally, (**D**) the collagen content was measured in bronchoalveolar lavage fluid (BALF) samples with a Sircol^TM^ assay. The experiment was repeated twice with similar results. All data are shown as mean ± SEM per group, * *p* < 0.05; ** *p* < 0.01; *** *p* < 0.001 by Mann–Whitney test.

The fibrogenesis observed in IPF is mostly a parenchyma remodeling effect. Therefore, whole lung section images were recorded to evaluate the amount of fibrogenesis in the lung parenchyma (Figure 2A—main image). Additionally, images of proximal (Figure 2A—small image) and distal bronchi (Figure S3) were made to evaluate the peribronchial deposition of collagen. Lung sections were stained with Sirius red for these analyses. We observed that the collagen expression and deposition in the parenchyma (Figure 2B) and around proximal (Figure 2C) and distal (Figure S3) bronchi were downregulated in KO mice compared with WT mice after bleomycin-induced lung fibrosis.

Moreover, a higher presence of proteins, predominately collagen fibers, was detected in the BALFs of both KO and WT mice after bleomycin administration (Figure 2D). Here, a clear reduction of the collagen content was observed in KO mice in comparison with WT mice upon bleomycin administration.

### 2.3. Airway Smooth Muscle Contributes to Asma^+^ Cell Accumulation in the Parenchyma and to Its Own Hyperplasia through Wnt5a Expression

**Figure 3:**
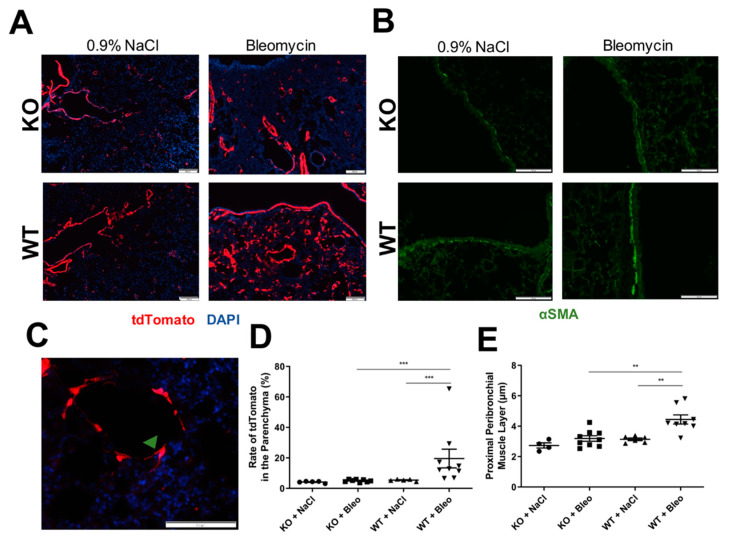
Histological analysis of αSMA-positive cells. (**A**) Expression of reporter fluorescence protein tdTomato (red) in αSMA positive cells was analyzed in fixed frozen sections of lung with 4% PFA and covered with medium DAPI (blue); images were recorded (scale: 200 µm). (**B**) The peribronchial muscle layer of proximal bronchi was stained with antibody against αSMA and a secondary antibody coupled with AF488 in paraffin-embedded lung sections (scale: 100 µm). (**C**) Representation of an αSMA positive cell (green arrow) with typical spindle-shape and dendritic morphology of myofibroblasts (scale: 100 µm). Both immunostainings were evaluated with the help of ImageJ software, (**D**) rate of tdTomato expressing cells were evaluated by recording at least eight images of the parenchyma, where airways and blood vessels were avoided. The total area of cells expressing tdTomato (red) as a percentage was obtained and calculated referring to the total area of cells stained with DAPI (blue) as a percentage: Rate = (% Area expressing tdTomato × 100)/% Area expressing DAPI. (**E**) The thickness of the peribronchial muscle layer was obtained by measuring five random pictures of proximal bronchi per mouse. All data are shown as mean ± SEM per group, ** *p* < 0.01; *** *p* < 0.001 by Mann–Whitney test.

Both mouse lines (KO and WT) expressed the fluorescent protein tdTomato as the reporter gene under the αSMA promotor, therefore, expression was used to detect SMC and myofibroblasts in situ. Consequently, frozen sections of lung tissue were fixed and analyzed (Figure 3A,C). It was possible to observe a very low number of αSMA^+^ cells around the parenchyma of the control mice, but also in bleomycin-treated KO mice. However, a higher number of αSMA^+^ cells was observed in bleomycin-treated WT mice (Figure 3A). Images of the parenchyma were recorded and airways and blood vessels (SMC) were avoided due to the higher expression of tdTomato around them. We could observe an increase of the number of αSMA^+^ cells in WT mice, which was not seen in the KO mice (Figure 3C). These αSMA^+^ cells present in the WT mice upon bleomycin-induced lung fibrosis resembled myofibroblasts due to their spindle-shaped and dendritic morphology [21] (Figure 3B).

Moreover, paraffin-embedded lung sections were stained with anti-αSMA (1A4 clone) and images of proximal bronchi (Figure 3D) were recorded to confirm the presence of the peribronchial smooth muscle layer and the potential effects of the KO in it. Here, we saw a clear thickening of the airway smooth muscle in the proximal bronchi in bleomycin-induced lung fibrosis, which was not observed in KO mice (Figure 3E).

### 2.4. Deletion of the Wnt5a Gene in Airway Smooth Muscle Cells Does Not Affect Leukocyte Infiltration into the Airways but Has Mixed Effects on Cytokine Expression

**Figure 4:**
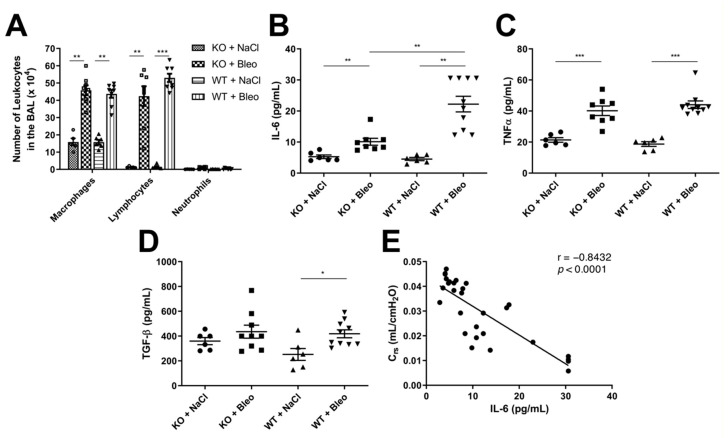
Analysis of inflammatory response upon bleomycin-induced lung fibrosis in KO and WT mice. (**A**) Total number of macrophages, lymphocytes and neutrophils in BALF samples from KO treated with saline solution (column filled with small squares) and bleomycin (bigger squares) and WT mice with saline (horizontal lines) or bleomycin inoculation (vertical line). The same lung lobe from each mouse was removed and homogenized in PBS containing proteinase inhibitors, (**B**) IL-6, (**C**) TNFα and (**D**) TGF-β were measured in the lung homogenate with ELISA and are represented in pg/mL in each graph. (**E**) Due to the high increase of IL-6 in WT compared to KO mice, IL-6 was correlated with compliance of the respiratory system (C_rs_) obtained from the lung function (Figure S2C). The experiment was repeated twice with similar results. All data are shown as mean ± SEM per group, * *p* < 0.05; ** *p* < 0.01; *** *p* < 0.001 by Mann–Whitney test.

The leukocyte extravasation and expression of proinflammatory and profibrotic cytokines were analyzed to determine whether Wnt5a KO has an influence on the bleomycin-induced inflammation (Figure 4).

The extravasation of leukocytes was increased upon bleomycin-induced lung fibrosis with a high number of macrophages and lymphocytes even after three weeks in the fibrotic phase (Figure 4A). A ratio of 1:1 of macrophages and lymphocytes in both WT and KO mice was observed upon bleomycin administration, but no differences were observed between the WT and KO mice in the fibrotic phase after three weeks, nor in the inflammatory phase after eight days (Figure S4C,F). Even though there were some outliers in WT mice that were treated with bleomycin in the inflammatory phase (Figure S4C), we decided to repeat the experiment with bigger groups (ten mice per group) and analyzed the different leukocytes in the bronchoalveolar lavage fluid with flow cytometry. No difference was observed in the flow cytometry analysis between WT and KO mice after bleomycin-induced lung fibrosis after eight days of intranasal administration (Figure S4F).

However, when we analyzed the cytokine expression, proinflammatory cytokines IL-6 (Figure 4B) and TNFα (Figure 4C) were upregulated upon bleomycin administration, but only IL-6 showed a higher expression in the WT mice than in KO mice (Figure 4B). Furthermore, the profibrotic cytokine TGF-β was analyzed and a slight upregulation in WT was observed upon bleomycin-induced lung fibrosis (Figure 4D). However, no significant difference was observed in KO mice with or without bleomycin administration, nor between the KO and WT. Expression of these cytokines was also equal in WT and KO mice in the inflammatory phase (Figure S4D,E).

Furthermore, an inverse correlation was observed in the expression of IL-6 with the compliance of the respiratory system (C_rs_) (Figure 4D), suggesting that higher IL-6 concentrations are observed in mice with increased stiffness of the lung tissue.

## 3. Discussion

We show here for the first time that Wnt5a KO in airway SMC leads to an improved clinical phenotype in a mouse model of bleomycin-induced lung fibrosis. These mice had a lower weight reduction and an improved overall score compared with wild-type mice. Moreover, they showed a reduced stiffness of the lung tissue as determined by the invasive lung function measurement. These overall improvements of the clinical parameters were based on a decrease of collagen deposition in the peribronchial areas and in the parenchyma of the lungs of KO mice in comparison with the WT mice. 

The reduced fibrosis could be a direct result of the Wnt5a role in the induction of proinflammatory cytokines through the NF-κB pathway [22]; however, no noticeable differences were observed in the number of trafficked leukocytes either in the inflammatory or in the fibrotic phase. Therefore, the decrease in collagen expression and deposition throughout the lung tissue is most probably a result of the Wnt5a role in ECM production and deposition [17], which could be affected by lower Wnt5a signaling through Fzd8 and Ryk receptors [17]. 

A higher number of αSMA-expressing cells was observed in the lung parenchyma of WT mice after bleomycin administration compared with the Wnt5a KO mice. Due to their spindle-like and dendritic morphology, it is likely that these cells are activated fibroblasts and myofibroblasts. These cells also express αSMA [23], as determined by the production of the reporter protein tdTomato. One important mechanism involved in lung fibrotic diseases is fibroblast proliferation and differentiation into myofibroblasts [24,25]. A study by Li et al. observed an impairment of myofibroblast differentiation and migration upon postnatal inactivation of Wnt5a in mice, moreover, this was also confirmed by inactivating the Wnt5a receptors Ror1 and Ror2 in fibroblasts [26]. These findings may suggest that airway smooth muscle-derived Wnt5a could play a role in fibroblast migration and differentiation in a bleomycin-induced fibrosis scenario. However, this study was done in 2-day-old mice, where alveologenesis is still occurring.

Martin-Medina et al. demonstrated that primary human lung fibroblasts are involved in Wnt5a signaling through extracellular vesicles that induce fibroblast proliferation and contribute to IPF pathogenesis [14]. Although it was shown that SMC from asthmatic patients can also be a source of Wnt5a [17], currently there are no reports that have analyzed the role of SMC derived Wnt5a in the development of lung fibrosis. Therefore, further studies are needed to evaluate the effect of airway smooth muscle-derived Wnt5a in the activation, migration and proliferation of fibroblasts in lung fibrotic diseases. 

Here, we investigated whether KO mice had any deviation in cytokine expression and found out that the expression of the proinflammatory cytokine IL-6 was at least half of that in WT mice after an induced lung fibrosis with bleomycin. On top of that, expression of the profibrotic cytokine TGF-β was not altered upon KO of Wnt5a, showing that the KO did not alter TGF-β production. TGF-β does not only stimulate Wnt5a expression, but it may act synergistically with Wnt5a to transform more cells into ECM-producing myofibroblasts as seen in SMC of asthmatics [17]. Therefore, even though TGF-β concentrations did not show a significant alteration, the absence of the synergism with Wnt5a lead to attenuated remodeling.

We also analyzed the effect of bleomycin in the inflammatory phase upon the KO of Wnt5a to understand better if Wnt5a could play a role in the inflammatory response that led to a worse fibrotic outcome. Therefore, we looked at the cytokine expression after eight days of bleomycin administration and found no alteration in the cytokine expression. Thus, the expression of Wnt5a in SMC of the airways does not affect the inflammation response upon bleomycin injury, but rather the production of ECM proteins that ultimately accelerate and aggravate the fibrogenesis of the lung, resulting in the high expression of the cytokine IL-6.

Zampetaki et al. studied the effect of biomechanical stress on IL-6 expression [27]. These authors showed that enhanced biomechanical stress in SMC leads to IL-6 production by activating the MAPK–NF-κB pathway and that other proinflammatory cytokines are not induced by it. For this reason, correlation analysis was performed with IL-6 expression and the lung function parameter, compliance (Crs). An inverse correlation was observed here, meaning that IL-6 concentration is higher in subjects where the mechanically forced airflow into and out of the lungs is more difficult. In other words, higher biomechanical stress upon breathing due to a higher deposition of ECM proteins could explain the higher expression of IL-6 in WT mice with lung fibrosis compared with the KO mice. This observation was also seen in other publications, where induced lung injury by mechanical ventilation in BalbC mice led to a higher IL-6 content in their BALFs [28]. 

Even though effect of Wnt5a in the NF-κB pathway is known [22,29], the knockout of Wnt5a did not show any noticeable changes in the inflammatory phase, as discussed before. Nevertheless, the activation of the MAPK–NF-κB pathway in SMC upon biomechanical stress [27] may be regulated by Wnt5a expression, which results in lower IL-6 expression in the fibrotic phase upon Wnt5a knockout as observed in our study.

Furthermore, the lower expression of IL-6 in KO mice could explain the ameliorated effect of the KO in the thickness of the airway smooth muscle layer, as it is known that the proinflammatory cytokine IL-6 is highly expressed in many lung diseases and can induce hypertrophy and hyperplasia in airway SMC [30,31]. 

Taking all the facts into consideration, we were able to demonstrate here that airway SMC can contribute to the fibrogenesis of the lung by expressing Wnt5a. We demonstrated by knocking out Wnt5a in airway SMC that Wnt5a released from these cells contributes to the expression and deposition of collagen throughout the lung, leading to higher fibrosis. The higher fibrogenesis of the lung and biomechanical stress upon breathing can lead to a higher production of the proinflammatory cytokine IL-6. We also demonstrated that airway SMC can suffer hypertrophy or hyperplasia upon bleomycin-induced lung fibrosis, which was abrogated upon the KO of the gene in these cells without affecting the TGF-β expression. One could also observe in an overexpressing model by Koopmans et al. that Wnt5a stimulates the expression of αSMA around the airways [32].

Our study also has some limitations. Even though airway SMC may produce IL-6 [27] and may have contributed to its high expression in bleomycin-treated WT mice, we could not determine the source of the IL-6 in this study. However, since the leukocyte infiltration and production of other proinflammatory cytokines does not differ between WT and KO mice, an influence of Wnt5a on the immune response is unlikely. 

Moreover, the insertion of the Cre recombinase gene into the αSMA promotor does not allow the expression of the enzyme only in SMC because it is also expressed in fibroblasts upon their activation and differentiation into myofibroblasts [23]. Thus, the Wnt5a gene may also become excised in this cell type. Even though reports show a bioavailability of tamoxifen to be much lower than seven days [33,34], it cannot be excluded that residual tamoxifen or metabolites are present during the activation and proliferation of fibroblasts after bleomycin administration. Therefore, further studies should address the question whether myofibroblasts in fibrotic foci also contribute to the fibrotic phenotype by the production of Wnt5a.

## 4. Materials and Methods

The methods are summarized here; please see the Supplementary Materials for a more detailed description.

### 4.1. Animal Ethical Approval

All animal experiments were granted and supervised by the animal ethics committee at the State Agency for Nature, Environment and Consumer Protection North Rhine-Westphalia (LANUV, Recklinghausen, Germany) (identification code: 84-02.04.2016.A387 and date of approval: 31 December 2016).

### 4.2. Mice

iSma-Cre (C57BL6/J/iSma-Cre-ERT2/tdTomato) and Wnt5a^f/f^ mice (C57BL6/J background) were obtained from Saverio Bellusci from the University of Giessen, Giessen, Germany and Terry Yamaguchi from the National Cancer Institute, Churchville, MD, USA, respectively [35,36]. Mating between both lines were made to obtain Wnt5a KO mice (iSma-Cre/tdTomato/Wnt5a^f/f^) (Figure S1). Mice were housed in pathogen-free conditions and all animal experiments were performed according to ARRIVE guidelines. All mice used were male and between 11 weeks and 12 weeks old upon bleomycin inoculation. Mouse genotypes were determined by polymerase chain reaction with primers stated in the Supplementary Materials.

Mice were supervised daily with measurement of the weight and symptoms which were evaluated by giving a score to each mouse daily (Table S1). When a score of 20 was reached, the mice were euthanized. 

### 4.3. Bleomycin-Induced Fibrosis

**Figure 5:**
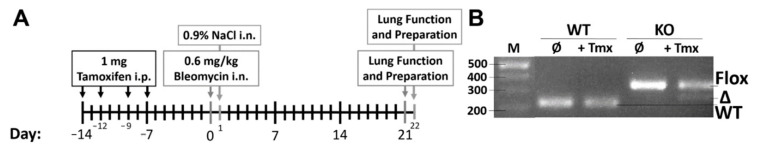
Experimental plan and confirmation of Wnt5a excision. The experiment was performed with four distinguishable groups: 1. Knockout (KO) + NaCl—Control Wnt5a KO mice that were treated with tamoxifen and saline solution i.n.; 2. KO + Bleo—Wnt5a KO mice that were given tamoxifen and lung fibrosis was induced with bleomycin; 3. WT + NaCl—Control reference mice (iSma-Cre) that were given only tamoxifen and saline solution i.n.; 4. WT + Bleo—Reference mice that were given tamoxifen and lung fibrosis was induced with bleomycin. Mice that only express Cre enzyme and tdTomato upon tamoxifen injection (iSma-Cre mice) were used as WT, due to no conditional KO of any gene. (**A**) All mice were treated with tamoxifen, lung fibrosis was induced with bleomycin in KO and WT mice on day 0, and control KO and WT mice treated with saline solution on day 1. Lung function measurement and mouse preparation was done exactly three weeks after the i.n. inoculation of bleomycin and saline solution. (**B**) Ear tissue from WT and KO mice was taken before and after tamoxifen administration to demonstrate the KO of the Wnt5a gene (268 bp) in KO mice and wild-type phenotype in WT mice (223 bp) after tamoxifen injection. The KO mice still had many cells where the gene was only floxed (351 bp), due to the higher number of αSMA negative cells.

Wnt5a KO mice were used against iSma-Cre mice (WT), with the latter ones being the reference as they represent normal physiological and morphologic conditions, except for the expression of the reporter gene tdTomato. All mice were treated with 10 mg/mL tamoxifen (T5648; Sigma-Aldrich, St. Louis, MO, USA) four times via i.p., each application of 100 µL was administered every 48/72 h. One week later, 50 µL of 0.6 mg/kg bleomycin sulfate (Cell Pharm, Hannover, Germany) was given once via i.n. and 50 µL of saline solution (0.9% NaCl) was given to the control groups. Mice were then sacrificed after three weeks of observation (Figure 5A).

### 4.4. Characterization of Wnt5a Knockout

The WT and KO mice were characterized to confirm Wnt5a excision in KO mice upon tamoxifen injection. Consequently, ear tissue was removed before and after the tamoxifen injection and DNA was isolated from the tissue with alkaline lysis solution (25 mM NaOH/0.2 mM EDTA-Sodium) for 10 min at 95 °C and subsequently neutralized (40 mM Tris-HCl). The DNA was amplified with the same primers for the Wnt5a genotype and analyzed on a 1.5% agarose gel (Figure 5B).

### 4.5. Lung Function

Mice were deeply anesthetized by using 162.5 mg/kg ketamine (CP-Pharma, Burgsdorf, Germany) and 22.1 mg/kg xylazine (BD Pharma, Franklin Lakes, NJ, USA), and an incision in the trachea was made to insert a cannula to connect to the flexiVent (SCIREQ, Montreal, QC, Canada). Different scans were made to evaluate the lung function (pressure–volume curve, single compartment and constant phase model) (for a better understanding of this technique, please read the article from Devos et al. [37]). 

### 4.6. Lung Samples and Analysis

Bronchoalveolar fluid (BALF) was obtained by instillation of the lungs with 1 mL PBS twice. The total number of leukocytes were counted by staining with Türk’s solution. The rest of the leukocytes were obtained by centrifugation and cytospun in slide sections for staining with HAEMA-fast staining (LT-Sys, Berlin, Germany). A differential count of leukocytes was done by counting a total number of 300 cells per slide. Furthermore, 200 µL of each BALF was used to quantify the collagen content with the Sircol^TM^ Collagen assay (Biocolor, Carrickfergus, UK), according to the manufacturer’s instructions.

The middle right lung lobe of each mouse was excised and washed with PBS to remove the blood. The lungs were homogenized in PBS with a complete protease inhibitor cocktail (#04693132001, Roche, Basel, Switzerland) using the gentleMACS Dissociator (Miltenyi Biotec, Bergisch Gladbach, Germany) to measure the cytokines with commercial ELISA kits.

### 4.7. Histological Analysis

The left and upper right lung lobes were fixed in 4% PFA. The upper right lobe was incubated overnight in 10% saccharose and subsequently in 30% saccharose before snap freezing into 10 µm frozen sections. The left upper lung was embedded in paraffin and cut into 4 µm sections. Paraffin-embedded sections were stained with Sirius red to analyze fibrogenesis and five different images of the proximal and distal bronchi were recorded. Images of whole lung sections were obtained with the Zeiss Axio Scan.Z1 and at least eight zoomed-in images of the parenchyma were recorded per mouse, avoiding airways and blood vessels. 

### 4.8. Immunofluorescence Staining and Detection

Tissue sections were deparaffinized and rehydrated, followed by antigen retrieval with 0.8% SDS for 10 min. Blocking and permeabilization was done by incubating sections in 10% fetal bovine serum and 0.5% TritonX-100 for 30 min. Sections were incubated for 90 min with mouse anti-αSMA primary antibody (A2547; Sigma-Aldrich, St. Louis, MO, USA) and 60 min with secondary goat antibody conjugated with AF488 (ab150141; abcam, Cambridge, UK). In order to detect the reporter fluorescent protein tdTomato in the lung tissue, frozen sections were fixed in 4% PFA for 5 min. Both cell nuclei from paraffin and the frozen sections were counterstained with Roti-Mount FluorCare DAPI (Roth, Karlsruhe, Germany). All steps were performed at room temperature.

### 4.9. Statistics

Each control group was composed of six mice, while ten mice were used per group in the bleomycin-induced lung fibrosis model. All quantitative data are represented by the mean and standard error of the mean (SEM). Column graphs were analyzed through independent comparison of two groups with the Mann–Whitney test, while pressure–volume curves were analyzed with a two-way ANOVA with the Bonferroni post-test. A *p* < 0.05 was considered statistically significant for all analyses.

## 5. Conclusions

IPF is a fatal disease with a constant decline of lung function. It is already known that TGF-**β** is one of the factors that drives the remodeling of the lung tissue. Here we show that airway smooth muscle cells can contribute to the development of lung fibrosis by expressing Wnt5a. Wnt5a increases the number of fibrotic foci in the lung leading to enhanced collagen deposition and increased stiffness of the lung tissue. Therefore, this study provides interesting new insights in the role of SMC-derived Wnt5a as a profibrotic factor in lung fibrosis, revealing it as a potential target besides that of TGF-β to modulate the progression of lung fibrosis. Thus, in further studies we will try to adapt our model to a more therapeutic setting, where we will knock out the Wnt5a gene when fibrosis is already established.

## Data Availability

Data is contained within the article and Supplementary Materials.

## Supplementary Materials

The following are available online at https://www.mdpi.com/article/10.3390/ph14080755/s1, Figure S1: Gene expression scheme of the KO mice used (Wnt5a KO), Figure S2: Lung function parameters obtained from the flexiVent system, Figure S3: Collagen expression in the distal peribronchial area, Figure S4: Analysis of the Wnt5a role in the inflammatory response after eight days of bleomycin-induced lung injury in KO and WT mice, Table S1: Score table used for mouse clinical symptoms.
