# Supplementary material for: The Pathogenic Role of Smooth Muscle Cell-Derived Wnt5a in a Murine Model of Lung Fibrosis
Source: Pharmaceuticals (Basel). 2021 Jul 31;14(8):755. doi: 10.3390/ph14080755 (PMC8401301; doi:10.3390/ph14080755)
Supplement: Supplementary file 1 [file pharmaceuticals-14-00755-s001.zip › pharmaceuticals-1303703-supplementary.pdf]

Supplementary material online

The pathogenic role of smooth muscle cell derived Wnt5A in a murine model of lung fibrosis

André Carmo-Fernandes[1], Michelle Puschkarow[2], Karin Peters[1], Stefanie Gnipp[2] and Marcus Peters[1,*]

1 Department of Molecular Immunology, Ruhr-University Bochum, 44780 Bochum, Germany

2 Department of Experimental Pneumology, Ruhr-University Bochum, 44780 Bochum, Germany

*Corresponding Author: Dr. Marcus Peters, AG Immunologie der Lunge, Abteilung für Molekulare Immunologie, Ruhr-University Bochum, Universitätsstraße 150, D-44801 Bochum, Email: Marcus.peters@rub.de

DETAILED METHODS

Genotyping

Following primers were used to genotype each mouse: 5'- GACTCAACAGCATTGAGACATGTTTGT-3', 5'- TCTCGGAAGTACTGCTATCTCCTACC-3' and 5'- GGCTCCCTCAGTCAGTGTTTTCA-3' to determine floxed Wnt5a, excised gene or WT (Fig. 1B), 5'- ATTTGCCTGCATTACCGGTC-3' and 5'- AGGCTTTTGTTTTGCAACTA-3' to confirm the presence of Cre enzyme and 5'- AAAGGAGCTGCAGTGGAGTA-3', 5'- CCGAAAATCTGTGGGAAGTC-3', 5'- CTGTTCCTGTACGGCATGG-3' and 5'- GGCATTAAAGCAGCGTATCC-3' to determine tdTomato gene sequence.

Bleomycin-induced fibrosis

Tamoxifen was diluted in corn oil (Sigma-Adrich, München, Germany) in a ratio of 1:10 from a 100 mg/mL stock solution (100 mg/mL tamoxifen in ethanol (VWER Chemicals, Radnor, USA)). Bleomycin was diluted in saline solution (0,9% NaCl).

To analyze the inflammatory phase of bleomycin same Wnt5a KO line (KO) and iSma-Cre (WT) mice were used, first with a small group of 4 KO and 5 WT mice and afterwards with 10 mice per group for Flow Cytometry analysis of the different leukocytes in the Bronchoalveolar lavage fluid. All of the mice were treated with tamoxifen and bleomycin as previous animal experiment, except that mice were sacrificed after eight days of bleomycin-induced fibrosis.

Characterization of Wnt5a knockout

DNA from ear tissue was isolated with 75 µL of alkaline lysis solution (25 mM NaOH / 0,2 mM EDTA-Sodium) for 10 min at 95°C and afterwards neutralized (40 mM Tris-HCl) with the same volume. DNA was amplified with Red HS Taq Master mix kit (Biozym, Hess. Oldendorf, Germany) and same primers for Wnt5a genotype were

used, all according to manufacturer's instructions. Samples were analyzed on a 1,5% agarose gel (Fig. 1B) along with a DNA marker (#10002, Biomol, Hamburg, Germany).

Lung samples and analysis

Bronchoalveolar fluid (BAL) was obtained by instillation of lungs with 1 mL PBS twice. 50 µL of the BAL sample was immediately stained with equal volumes of Türk's solution. Leukocytes from the 2 mL BAL samples were then centrifuged for 10 min at 3000 rpm and cells were resuspended in 100 µL PBS. Resuspended cells were cytospinned for 2 min at 600 rpm in slide sections and stained with HAEMA-fast staining (LT-Sys, Berlin, Germany).

Each lung was homogenized in 1,4 mL of PBS containing protease inhibitors. Each cOmplete Protease inhibitor cocktail tablet was solubilized in 50 mL PBS.

Lung function

0,8 mg/kg of Pancuronium (P1918; Sigma-Aldrich, München, Germany) was given via i.p. injection to each mouse before performing lung functions tests to avoid spontaneous breathing.

Histological Analysis

Sirius red staining was performed by melting the paraffin from paraffin-embedded sections in 90°C for 10 min and incubating afterwards in RotiHistol (Roth, Karlsruhe, Germany) for another 10 min. Tissue sections were rehydrated by differential serial dilution of ethanol and stained with Picrosirius red solution (0,1% Sirius red in 1,2% Picric acid) for 40 min. Excess of stain was washed for two times with 2% acetic acid by diving sections in and out for 2 min. Sections were then dehydrated and covered with Eutkitt (ORSAtec, Bobingen, Germany) and glass covers.

Peribronchial images were used to calculate thickness of collagen layer by measuring the thickness five times per image using ImageJ (NIH, Maryland, USA). The

percentage area of stained parenchyma tissue was done by splitting the color channels and measuring the percentage of red in each single picture with ImageJ.

Immunofluorescence Analysis

Five images from proximal and distal bronchi were recorded from paraffin-embedded sections stained for αSMA, thickness of αSMA layer per mouse was obtained by measuring the thickness of each image five times in different locations of the airway. The mean of each mouse was obtained by the mean of each picture obtained.

Five random images throughout the parenchyma of each mouse were recorded from fixed frozen sections for tdTomato expression and DAPI. Images were analyzed through ImageJ, by splitting color channels. The total area of cells expressing tdTomato (red) in percentage was obtained and calculated in reference of the total area of cells stained with DAPI (blue) in percentage: rate of tdTomato = (% Area expressing tdTomato*100) / % Area expressing DAPI.

Cytokine Analysis

Lung homogenates were used for the cytokine analysis, ELISA Kit Ready-SET-Go! was used to measure cytokines (IL-6, TNFa, TGF-β), according to the manufacturer's recommendations.

Briefly, a 96 Well plate was coated with 50 µL capturing antibody overnight at 4°C. On the next day, the wells were washed with PBS-T and blocked with assay diluent for at least 2 h. Standard and samples were diluted and a total of 50 µL was pipetted per well and incubated overnight at 4°C. The wells were washed with PBS-T and incubated with 50 µL of detecting antibody at room temperature for 1 h. After incubation, wells were washed again and incubated with 50 µL of the enzyme for 30 min. All wells were afterwards washed before applying 50 µL of the substrate (TMB) and let incubate for around 5 to 10 min in the dark. The reaction was stopped with 25 µL of 0,1 N sulphuric acid and absorbance was measured at 590 nm with a microplate reader.

Flow Cytometry

For the analysis of leukocytes in the Bronchoalveolar fluid in the inflammatory phase, 10 KO and 10 WT mice were used. Unspecific binding was blocked with CD16/32 (#553142, Becton Dickinson) and afterwards stained with the antibodies: CD45R APC(#17-0452-82, Thermo Fisher Scientific), Ia antigen AF700 (#56-5321-82, Thermo Fisher Scientific), Ly-6G FITC (#553126, Immunotools), CD4 Pacific Blue (#558116, Becton Dickinson), CD8 PE-Cy7 (#100722, Biolegend), CD11c eFluor506 (#69-0114-82, Thermo Fisher Scientific), CD45 PerCP/Cy5.5 (#45-0451-82, Thermo Fisher Scientific), TER-119 APC/eFluor780 (#47-5921-82, Thermo Fisher Scientific), CD11b PE/Cy5 (#15-0112-82, Thermo Fisher Scientific), CD3 PE (#50-0031-SU05, TonboBiosciences), SIGLEC F SuperBright600 (#56-1702-82, Thermo Fisher Scientific) and NK1.1 SuperBright702 (#67-5941-82, Thermo Fisher Scientific). All measures were done with CytoFlex LX (Beckman Coulter, USA) and analyzed with Kaluza 2.1.

Table S1: Score table used for mouse clinical symptoms.

Clinical Symptoms	Score
Weight	
Unaffected or increased	0
Weight loss 5-10%	1
Weight loss 11-20% in ≤5 days	5
Weight loss 11-20% in >5 day	10
Weight loss ≥20%	20
General Symptoms	
Smooth and shiny fur; Clean body orifices; Clear and shiny eyes	0
Defects in the fur (decreased or excessive body care)	1
Dull fur; Scruffy body orifices; Cloudy eyes; Increased muscle tone	5
Dirty fur; Sticky or moist body orifices; Cloudy eyes; High muscle tone	10
Cramping/Paralysis (hull muscles/extremities); Breathing noises; Hypothermia	20
Clinical Parameters	
Normal breathing; Warm extremities; Good blood circulation in body orifices	0
Slight deviation from normal conditions	1
Change of breathing rate (±30%) in ≤3 days	5
Change of breathing rate (±30%) in >3 days	10
Change of breathing rate (±50%)	20

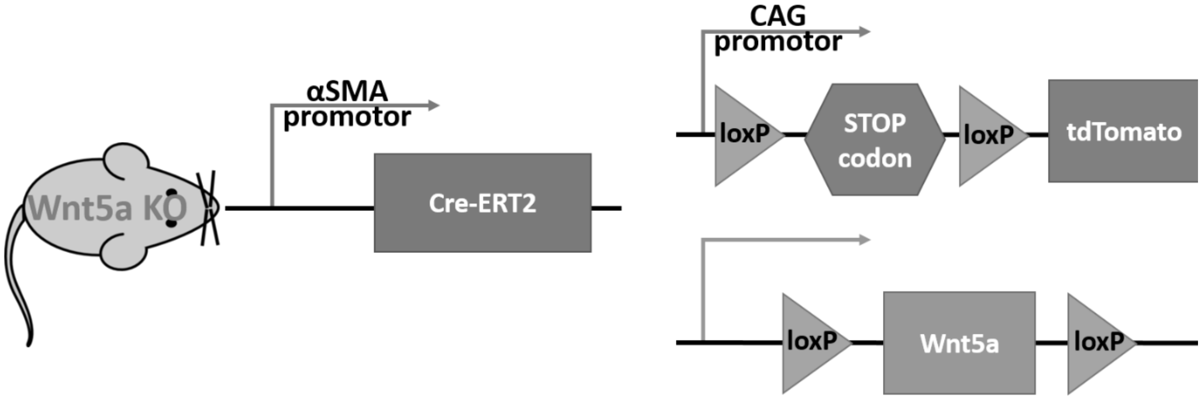

Figure S1 – Gene expression scheme of the KO mice used (Wnt5a KO). The gene for Cre recombinase conjugated with an estrogen receptor (ER^{T2}) is inserted in the αSMA promotor, thus is only expressed in αSMA positive cells. After injection of tamoxifen, Cre-ER^{T2} can bind to it and translocate to the nucleus to excise the floxed stop codon after the CAG promoter in ROSA26 locus and the floxed Wnt5a gene.

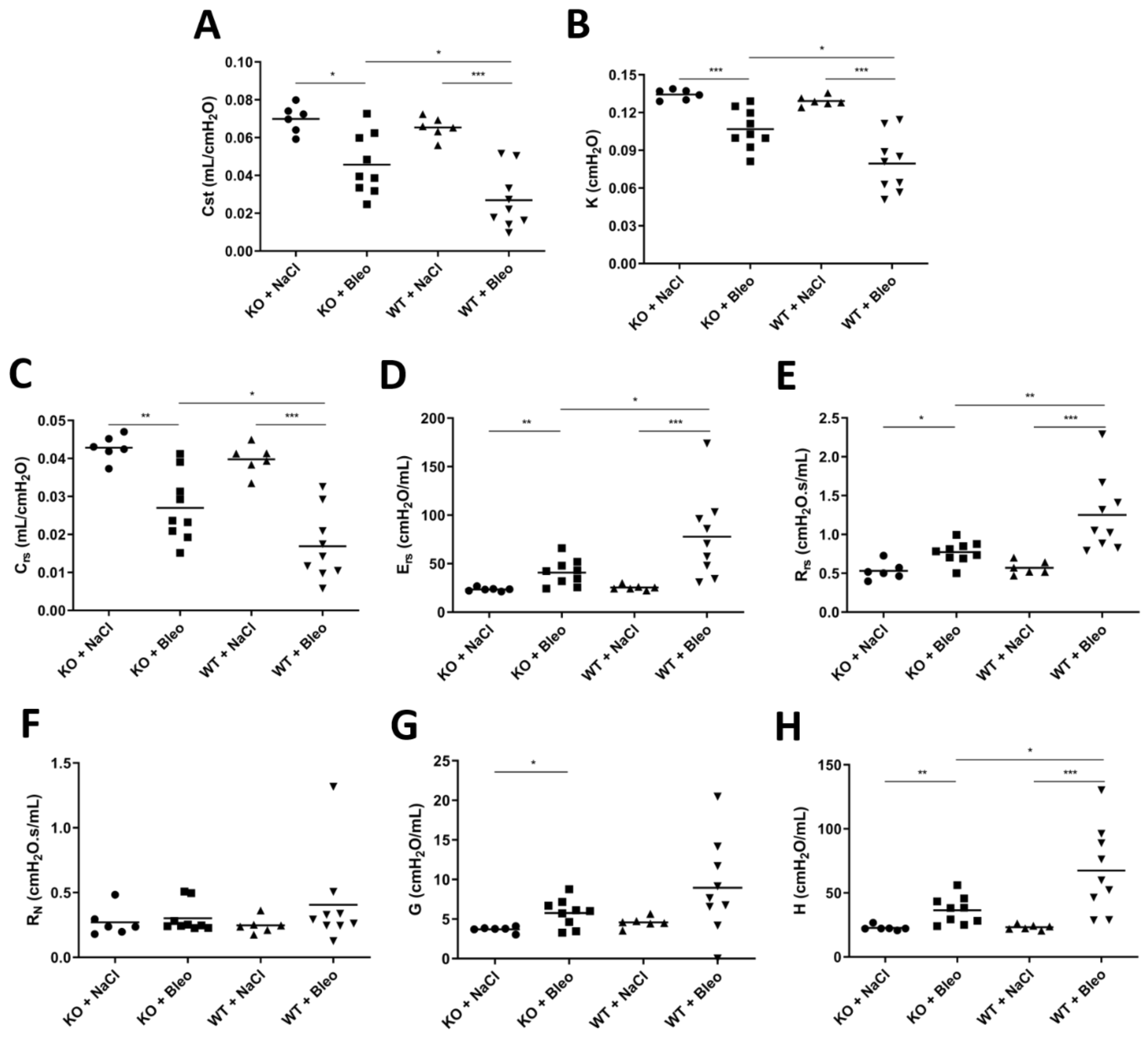

Figure S2 – Lung function parameters obtained from the flexiVent system. Besides the inspiratory capacity volume measured from the PV curve, **(A)** static compliance and **(B)** shape constant were obtained from the curve, which represents the intrinsic elastic properties of the respiratory system and indicates the phenotype of the chronic disease, respectively. Both parameters decline in case of fibrotic lung diseases. A scan based on the single compartment model was also done and allowed to determine the following parameters of the respiratory system: **(C)** compliance – how easy the lungs can be inflated, **(D)** elastance – elastic properties that the airflow needs to overcome to reach the tidal breathing of each mouse, and **(E)** resistance – resistive forces against the airflow in and out the lungs. The model of constant phase was used to **(F)** analyze the effect of bleomycin in the resistance against the airflow in the main conducting airways – Newtonian resistance, **(G)** the ability of the lung to retract and revert to the original shape upon forced airflow – tissue elastance, and **(H)** the energy lost within the peripheral airways due to resistance – tissue damping. Mean data are represented here.

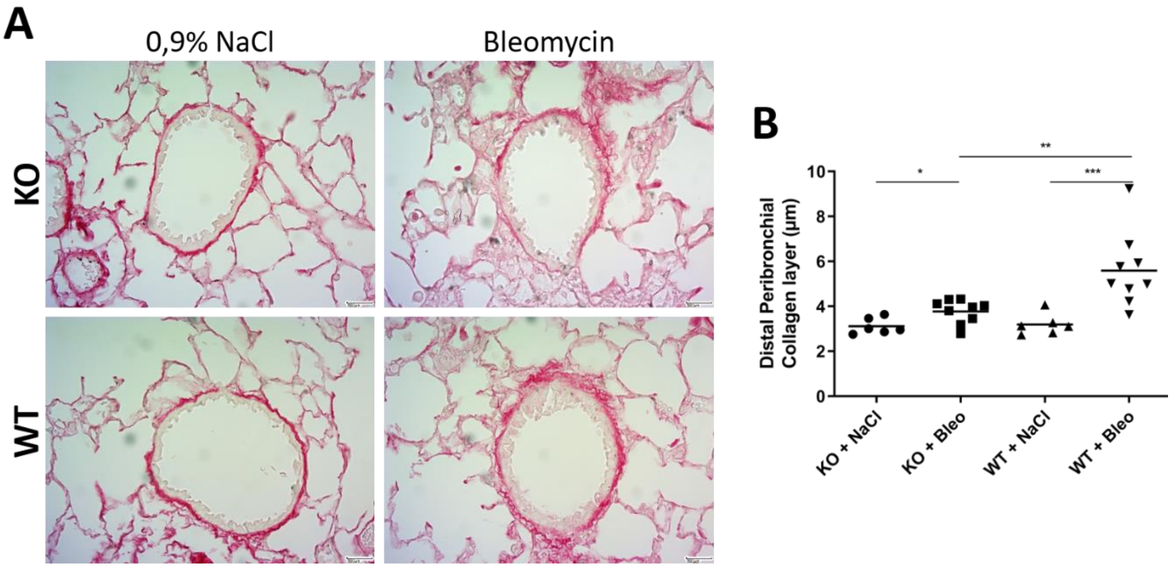

Figure S3 – Collagen expression in distal peribronchial area. (A) Images were recorded of distal bronchi (scale: 500 µm) stained with Sirius red, where **(B)** the thickness of collagen layer in the peribronchial area was measured in µm. Mean data are represented here.

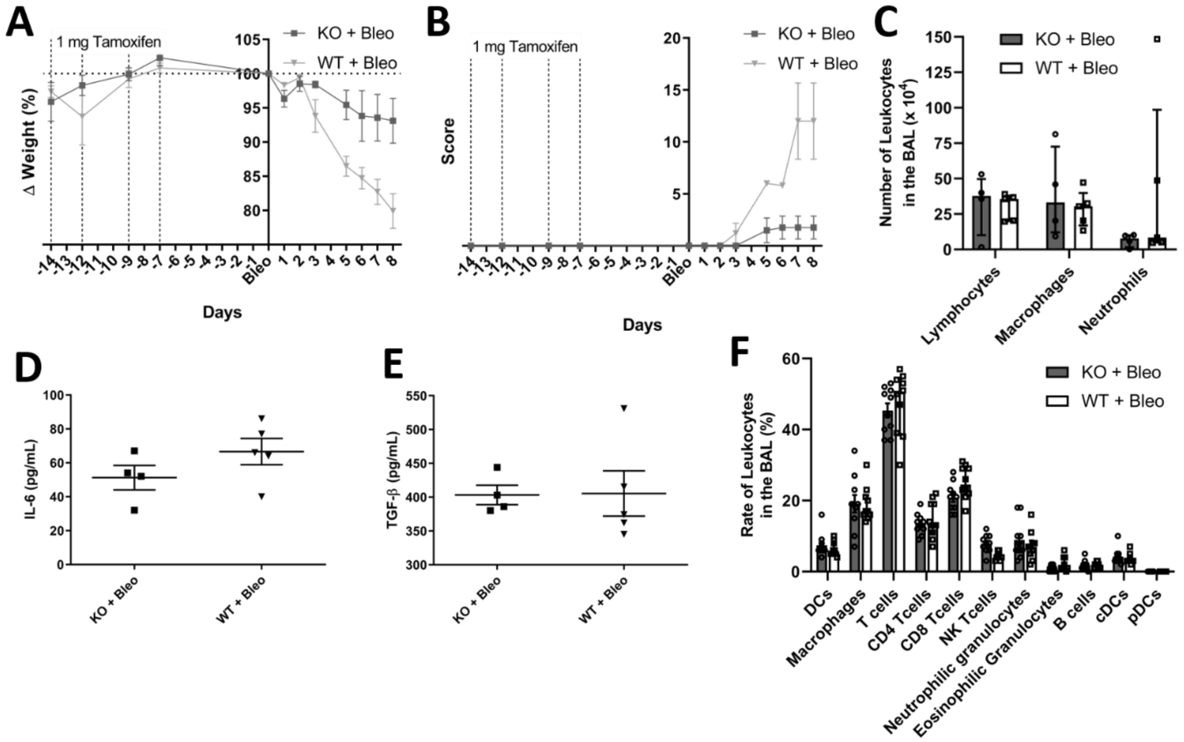

Figure S4 – Analysis of Wnt5a role in the inflammatory response after eight days of bleomycin-induced lung injury in KO and WT mice. For this analysis, KO and WT mice were used to induce lung fibrosis with bleomycin, however, mice were sacrificed eight days after bleomycin to analyze cytokine expression upon induced inflammation with bleomycin. **(A)** Weight variation and **(B)** score evaluation of both mice showed the same tendency of worse outcome in WT than in Wnt5a KO mice after bleomycin-induced lung fibrosis. **(C)** Total number of macrophages, lymphocytes and neutrophils in Bronchoalveolar lavage fluids of both bleomycin-treated KO (n=4; Grey column) and WT (n=5; White column). Expression of pro-inflammatory cytokines **(D)** IL-6 and **(E)** TGF-β were measured in the lung homogenate and no difference was seen between KO and WT mice. TNFα was also measured in both groups, but there was no detectable amount of the cytokine in the lung homogenate samples. **(F)** Rate of different leukocytes in Bronchoalveolar lavage fluids from both KO (n=10; Grey column) and WT (n=10; White column) mice analyzed by flow cytometry to confirm previous results. Data are presented as mean ± SEM (**A**, **B**, **D** and **E**) or in median ± interquartile range (**C** and **F**).
